# Deep-Learning-Enabled SEM Image Segmentation Coupled with Laser Confocal Raman Microscopy: Elucidating Microstructure and Drug Spatial Distribution in Leuprorelin Acetate Microspheres

**DOI:** 10.3390/ph19060948

**Published:** 2026-06-16

**Authors:** Wei Zhang, Zhihong Xu, Li Jiang, Xiaohu Tang, Chao Wang, Aiping Wang, Wanhui Liu

**Affiliations:** 1 Innovation Center of Advanced Drug Delivery System and Biotech Drugs in Universities of Shandong, Key Laboratory of Molecular Pharmacology and Drug Evaluation, Ministry of Education, School of Pharmacy, Yantai University, Yantai 264005, China; zhangwei125@nifdc.org.cn (W.Z.); xuzhihongrd@luye.com (Z.X.); gwtz2026@163.com (L.J.); 2 National Institutes for Food and Drug Control, Beijing 102629, China; 3 State Key Laboratory of Advanced Drug Delivery and Release Systems, Shandong Luye Pharmaceutical Co., Ltd., Yantai 264003, China; tangxiaohu@luye.com (X.T.); wangchao@luye.com (C.W.)

**Keywords:** microspheres, scanning electron microscopy, deep learning image segmentation, pore structure, laser confocal Raman spectroscopy, drug distribution

## Abstract

**Background/Objectives**: The precise characterization of the key microstructural and physicochemical attributes in sustained-release microspheres remains a technical bottleneck, hindering the understanding of drug release mechanisms, and limiting insights into the “process–structure–performance” relationship. To address this, we developed novel methods to conduct in-depth research on the microscopic properties of microspheres. **Methods**: Scanning electron microscopy (SEM) combined with a deep learning-based image segmentation (DLIS) algorithm was established for quantitative analysis of the pore structure. Laser confocal Raman spectroscopy (LCRS) was employed for in situ, non-destructive, three-dimensional (3D) visualization and quantitative mapping of the active pharmaceutical ingredient (API) distribution within microspheres. **Results**: This study successfully developed and applied SEM-DLIS and LCRS as reliable tools for microstructural and physicochemical characterization. SEM-DLIS analysis revealed significant differences in surface and internal pore structure among microspheres from different manufacturers and between particles of different sizes from the same batch. LCRS imaging further identified distinct API distribution patterns: uniform dispersion, outer-layer enrichment, and heterogeneous distribution. The combined data elucidate that the initial burst release is governed by the synergistic effect of surface porosity and API surface enrichment, whereas the sustained release kinetics are jointly regulated by the internal pore structure, particle size, and API spatial distribution. **Conclusions**: The findings establish that microstructure dictates release behavior and that all observed structural variations are linked to critical process parameters (CPPs), validating the “process determines structure” hypothesis. The established methodology provides a critical technical framework for the reverse engineering and quality equivalence assessment of generic microspheres, as well as for the quality-by-design-based optimization of innovative drug products, thereby advancing both pharmaceutical development and regulatory science.

## 1. Introduction

Poly (lactic acid)/poly (lactic-co-glycolic acid) (PLA/PLGA) sustained-release microsphere formulations represent a mature drug delivery platform, leveraging their biodegradable properties and ability to provide sustained drug release over weeks to months [1,2,3]. They play a critical role in improving patient medication adherence and managing chronic diseases [4,5]. To date, more than 20 such formulations have been approved by the Food and Drug Administration (FDA) and the European Medicines Agency (EMA) for marketing [6]. However, there have been no approved generic versions despite the expiration of patents for some microsphere products. This is largely attributable to the unique micron-scale structure and complex drug release mechanisms of these formulations, which pose significant challenges for generic development [7].

The manufacturing process for these microspheres is highly complex, often lacking precise process control and real-time monitoring. The quantitative relationship between CPPs and critical quality attributes (CQAs) remains insufficiently defined, leading to considerable batch-to-batch variability during scale-up and difficulty in precisely controlling the initial burst release [8]. Furthermore, the current quality control system is inadequate, particularly in standardizing the assessment of key microstructural attributes such as internal architecture and spatial drug distribution [6,9].

These microstructural attributes are directly influenced by CPPs and production controls; even minor variations in process parameters can alter these characteristics, subsequently affecting drug release profiles and therapeutic outcomes. Therefore, in-depth investigation and systematic characterization of the microstructural and physicochemical properties of these formulations are crucial for the development and evaluation of generic drugs, while also providing critical insights for the precise design and quality control of innovative drug products.

Currently, the characterization of CQAs for PLA/PLGA microspheres is largely limited to parameters such as particle size distribution, polymer molecular weight distribution, encapsulation efficiency, drug loading, and in vitro release profiles [10,11]. However, both internal and surface porosity (including pore size and structure) represent key quality attributes that genuinely reflect CPPs and govern in vitro and in vivo release kinetics. Established methods for measuring microsphere porosity include SEM, flow porosimetry, mercury intrusion porosimetry, gas pycnometry, gas adsorption, and micro-computed tomography (CT) [12]. However, these methods have notable limitations. For instance, SEM typically provides only the qualitative assessment of morphological parameters. Mercury intrusion porosimetry is destructive and requires toxic reagents. Gas pycnometry and adsorption methods can introduce significant errors in calculations for the internal porosity of micron-scale microspheres. CT, while capable of layer-by-layer scanning of internal microstructures, has a maximum resolution of only about 1–2 μm, requires specialized equipment setup, and involves high research costs. As demonstrated by Sun et al. [13] in their study on isoperidone-loaded PLGA microspheres, SEM has primarily been utilized for the qualitative morphological observation of microsphere surfaces and cross-sections, relying on visual inspection to determine the presence of pores and the influence of process parameters. Similarly, research by Le et al. [14] and Zhai et al. [15] predominantly relied on SEM imagery for qualitative classification (e.g., distinguishing between a ‘porous core’ and a ‘dense shell’). However, such visual assessment methods fall short in providing precise quantitative analyses of porosity, pore size distribution, and pore area percentage. Consequently, microstructural differences between batches or formulations remain difficult to objectively quantify. Moving beyond the subjective visual inspection of SEM images, we employed an SEM-DLIS algorithm. This approach enables the automatic identification and quantification of internal microsphere attributes, specifically porosity, pore size distribution, and pore area proportion. Such quantitative analysis transforms previously ambiguous “microstructural differences” into precise, statistically comparable metrics.

Drug distribution represents another critical parameter for characterizing the microstructural and physicochemical properties of microspheres. Currently, there is a lack of universal tools for characterizing the spatial distribution of drugs within microspheres. In recent years, transition temperature microscopy (TTM) has been applied to determine the distribution of drugs and polymers by detecting the transition temperature across a single microsphere cross-section [16,17]. However, this technique requires a melting point difference of over 40 °C between the drug and polymer to generate a distinct TTM map. Y. Xue et al. employed SEM combined with energy-dispersive X-ray spectroscopy (EDS) to study the chemical composition distribution on a specific plane of microspheres [18]. Sample preparation first requires obtaining a flat cross-section through mechanical cutting/polishing/fracturing, which can alter the original morphology of the microsphere cross-section and only provides random sectional chemical composition data. Furthermore, synchrotron radiation CT has been used to analyze substance distribution within tablets [19]. A critical limitation when applying it to microspheres with diameters of 1–1000 μm is spatial resolution, with the highest achievable resolution being only 1–2 μm, which is insufficient for a detailed investigation of the spatial distribution of the API inside microspheres. While traditional confocal laser scanning microscopy can provide certain spatial information, it relies heavily on fluorescent labeling [15]. This not only risks altering the native physical state of the drug, but also faces challenges of spectral overlap in multi-component complex systems, thereby failing to achieve true “label-free” chemical specificity in imaging.

In this study, we utilized LCRS to achieve label-free, chemically specific 3D imaging of the internal drug distribution within microspheres, addressing a critical gap in non-destructive microstructural analysis where traditional methods often require exogenous labeling. LCRS is well-suited for microsphere analysis, offering a spatial resolution of approximately 0.3 μm (a 532 nm laser, an 1800 gr/mm diffraction grating, a 100× objective lens (numerical aperture, NA = 0.9), and a 50 μm confocal hole) and a physical stage resolution of 0.05 μm [20,21]. This technique has also been used to characterize tablet coatings [22]. However, that study required sample sectioning and was limited by the sample’s opacity to the laser. A key advantage of LCRS for analyzing PLA/PLGA-based microspheres is their optical transparency within the analytical field of view, analogous to “*glass beads*”, allowing lasers of different wavelengths to penetrate without attenuation. This transparency eliminates the need for physical sectioning or chemical modification, which can alter the sample’s native state. As highlighted by Wang et al. [23] in their comprehensive review, LCRS serves as a pivotal tool for in situ drug distribution analysis. By employing this combined strategy, we were able not only to precisely map the spatial coordinates of the drug (e.g., leuprorelin) within the microspheres, but also to correlate this distribution with pore network connectivity. This integrated approach enabled the elucidation of release mechanisms that were previously inaccessible to conventional methods.

This characterization framework, integrating deep learning-based quantitative segmentation with LCRS chemical imaging, exhibits broad generality across materials science. Its essence lies in addressing the core materials science question of how spatial architecture governs transport and response. As emphasized by Grossman and McNeil [24], the efficacy of nanotechnology in cancer medicine relies heavily on the precise control of nanostructures. Within this framework, our methodology can be directly translated to the fields of magnetic theragnostics and separation by quantifying the aggregation state and spatial distribution of magnetic nanoparticles within a matrix (analogous to our analysis of drug distribution), where one can optimize heating efficiency under alternating magnetic fields or migration behavior in external fields [25]. Furthermore, Melnikov et al. noted that the performance of magnetoimpedance sensors for detecting stray fields of magnetic particles in blood vessels inherently depends on the distribution density and connectivity of the magnetic phase at the interface—parameters precisely captured by the pore connectivity indices and spatial distribution metrics established in this study [26]. In biosensing applications, the pore topology and probe distribution at the sensor interface directly dictate detection sensitivity and response limits. Consequently, this study not only resolves specific quality control challenges for PLGA microspheres, but also provides a versatile, high-precision analytical paradigm for the microstructural characterization of diverse porous functional materials, nanocomposites, and integrated theragnostic formulations.

This study focuses on leuprorelin acetate microsphere formulations, including the patented formulation and two modified versions. These formulations are prepared using an emulsion solvent evaporation process, resulting in microporous structures on their surface and internally [27,28]. Due to differences in CPPs, they exhibit different particle size distributions and in vitro release profiles. The objectives of this study are: (1) to characterize the microstructure and chemical properties of the microspheres, including surface and cross-sectional porosity, pore size distribution, and pore area proportion, using SEM-DLIS; (2) to investigate the internal spatial drug distribution and generate 3D imaging via LCRS; (3) to elucidate the relationship between their manufacturing process variations and in vitro release behavior.

## 2. Results

### 2.1. Characterization of Leuprorelin Acetate Microspheres

**Figure 1:**
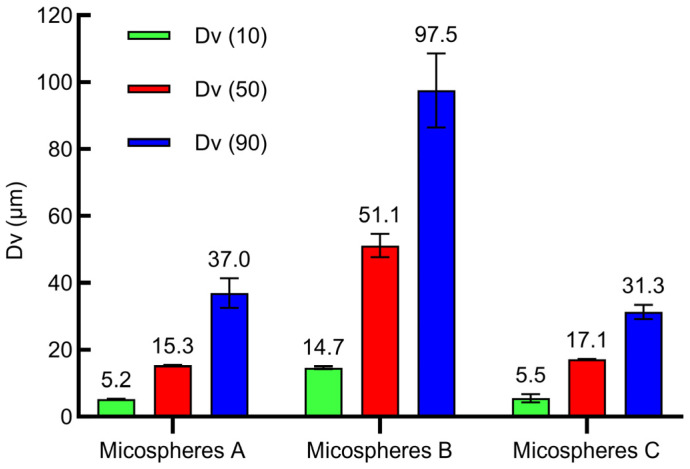
Particle size distribution of the microspheres.

The drug loading and particle size distribution of the leuprorelin acetate microspheres are summarized in Table 1 (Table A1) and Figure 1. The results indicate that the drug-loading capacity was comparable across the different manufacturers, at approximately 8%. In contrast, notable differences were observed in their particle size distributions. Microspheres A (Dv (50) = 15.3 μm, Dv refers to volume-based particle size distribution, and Dv (50) is the volume median diameter) and C (Dv (50) = 17.1 μm) exhibited similar median diameters, while Microspheres B was markedly larger (Dv (50) = 51.1 μm).

**Figure 2:**
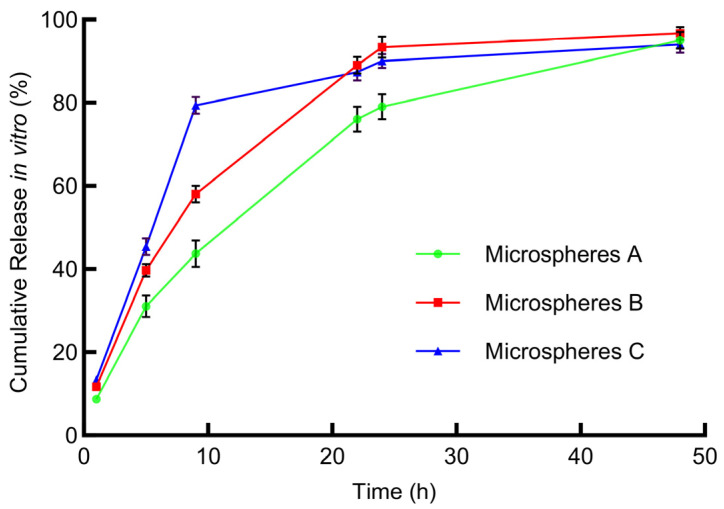
In vitro release profiles of leuprorelin acetate microspheres.

Based on the in vitro drug release profile in Figure 2 (Table A2), the formulations from the three manufacturers exhibited distinct release kinetics. Microspheres A showed the slowest and most gradual release profile, reaching about 44% at 9 h with the initial release about 9% at 1 h, while Microspheres C demonstrated the most rapid initial release, achieving approximately 79% cumulative release at 9 h with the initial release about 13% at 1 h. Microspheres B displayed an intermediate release rate, with approximately 58% release at 9 h, reflecting a balanced initial and sustained release behavior. Despite these early-phase differences, all three formulations approached near-complete release (~100%) by 48 h.

### 2.2. Morphology of the Microspheres

**Figure 3:**
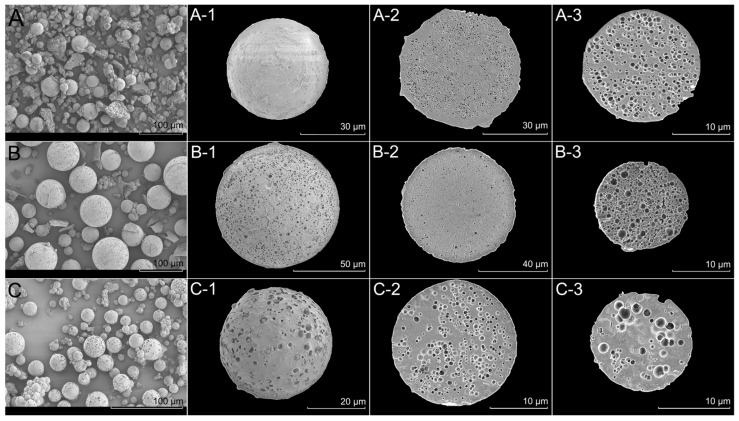
The external morphology of microspheres. Microspheres A (**A**,**A-1**). Microspheres B (**B**,**B-1**). Microspheres C (**C**,**C-1**). The internal morphology of microspheres. Microspheres A (**A-2**,**A-3**). Microspheres B (**B-2**,**B-3**). Microspheres C (**C-2**,**C-3**).

We performed comprehensive morphological analysis, including the overall appearance, surface, and cross-sectional structures, of the microspheres produced by three different manufacturers using SEM, as shown in Figure 3. The SEM images revealed that the microspheres from all three manufacturers were near-spherical particles with varying size distributions, and their surface and internal morphologies showed significant differences. Specifically, Microspheres A exhibited a smooth surface with indiscernible pores, while the cross-sections revealed a uniformly distributed internal porous structure. In contrast, Microspheres B displayed a smooth surface with uniformly distributed, dense pores, a characteristic also observed in the cross-sectional view. Interestingly, Microspheres C presented a rough surface with uneven, non-uniform pores. Notably, some pores contained embedded, irregularly-shaped particles at the micrometer scale. Unlike Microspheres A and B, cross-sections of different Microspheres C also revealed non-uniformly distributed and variably sized pores.

### 2.3. Porosity, Pore Size Distribution and Proportion of Pore Area

#### 2.3.1. Cross-Section Analysis

SEM images of the microspheres’ cross-sections were captured at various magnifications. The images were resolution-standardized and normalized prior to analysis. Image segmentation was then performed by processing the images through the pre-trained U-Net model, which automatically classified each pixel as either “pore” or “matrix”. Based on the segmentation results, the porosity was calculated as the ratio of the pore area to the total analyzed area, according to the following equation:

(1) $$Porosity=\frac{N_{void}}{N_{total}}\times 100\%$$

where *N_void_* is the number of pixels classified as pores, and *N_total_* is the total number of valid pixels in the image. The calculation results are presented in Table 2.

#### 2.3.2. Spherical Surface Analysis

To correct for surface curvature, a geometric model was applied. First, a pre-trained deep convolutional neural network (CNN), such as U-Net or DeepLabV3+, was used to perform semantic segmentation on the SEM images of the microspheres. This process classified each pixel as either “matrix” or “pore” and recorded its coordinates. Subsequently, the segmented contour of the microspheres was assumed to approximate a complete sphere or a spherical cap. A circle-fitting operation was performed on this contour to estimate the sphere’s center coordinates and radius (*R*). The final corrected porosity was then calculated using the following equation:

(2) $$Porosity=\frac{\sum_{i=1}^{N}\frac{A_{pixel}}{cos(\theta_{i})}}{4\pi R^{2}}\times 100\%$$

where *N* is the total number of pixels identified as pores, *A_pixel_* is the actual physical area of a single pixel (converted from SEM resolution), and *θ_i_* is the angle between each pore pixel and the observation axis (i.e., the normal to the image plane). The calculation results are presented in Table 2.

#### 2.3.3. Proportion of Pore Area

To quantify the spatial uniformity of pore distribution within the microspheres’ cross-sections, a statistical analysis of regional variance was performed. The cross-sectional area was divided into three regions of equal area (a1, a2, a3). The proportion of pore area (*X_i_*) was calculated for each region *i*. Given the limited sample size (n = 3), the arithmetic mean ($\bar{X}$) was used as the best estimator of the true average pore area proportion, calculated according to Equation (3). The standard deviation (*S*) of the pore area proportion across the three regions was calculated using Equation (4) to measure absolute variability. To objectively assess the relative dispersion of the pore distribution independent of the mean, the coefficient of variation (*CV*), defined as the ratio of the standard deviation to the mean, was calculated according to Equation (5). Finally, a metric for “uniformity” (*Hs*) was defined, representing the degree to which the measured values in each region approach the overall mean, which was calculated according to Equation (6). *Hs* and *CV* are inverse expressions of the same concept: a high uniformity value corresponds to a low dispersion value, indicating an equivalent proportion of pore area across the different regions, and consequently, a more homogeneous pore distribution. The calculation results are presented in Table 2.

(3) $$\bar{X}=\frac{1}{n}\sum_{i=1}^{n}\bar{X_{i}}$$

(4) $$S=\sqrt{\frac{1}{n-1}\sum_{i=1}^{n}{\left(X_{i}-\bar{X}\right)}^{2}}$$

(5) $$CV=\frac{S}{\bar{X}}\times 100\%$$

(6) $$H_{s}=100\%-CV$$

#### 2.3.4. Pore Size Distribution

The pore size distribution was determined by statistical analysis of the pore diameter data collected from the surface and cross-sections of the microspheres using GraphPad Prism software (v10.1.2, GraphPad Software Inc., San Diego, CA, USA).

**Figure 4:**
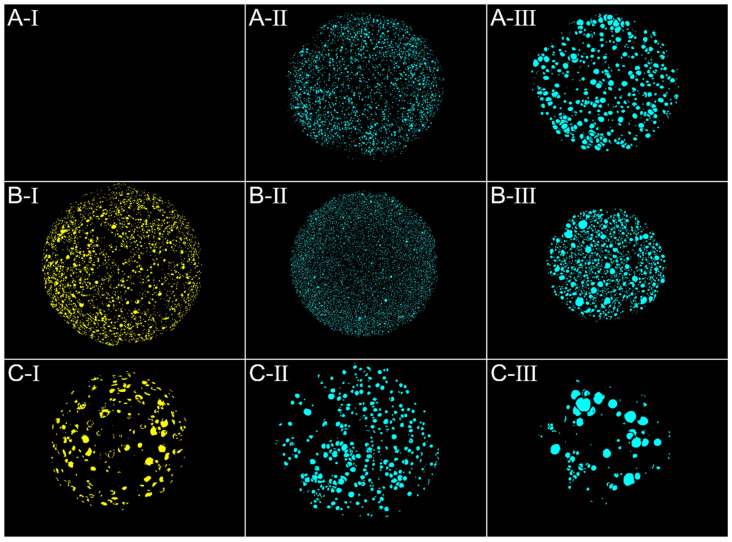
Identification of surface and cross-sectional pores. Microspheres A (**A-I**–**A-III**). Microspheres B (**B-I**–**B-III**). Microspheres C (**C-I**–**C-III**).

**Figure 5:**
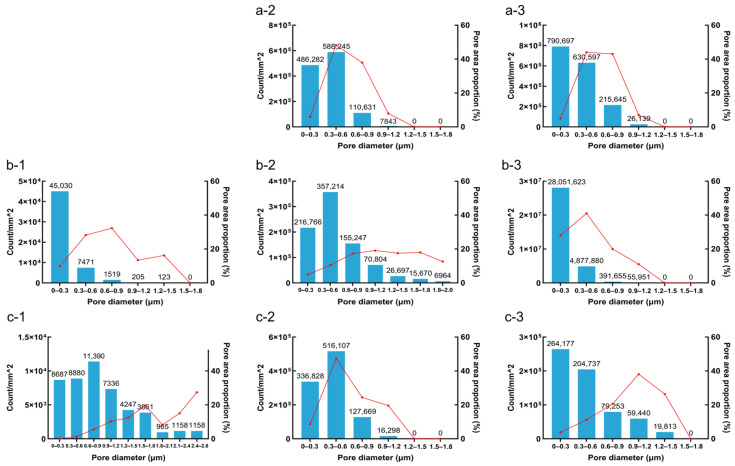
Pore number, pore size distribution, and pore area proportion. Microspheres A (**a-2**,**a-3**). Microspheres B (**b-1**–**b-3**). Microspheres C (**c-1**–**c-3**).

**Figure 6:**
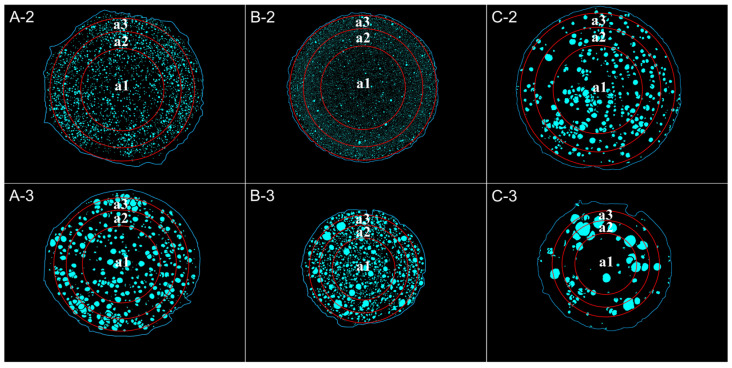
Division of cross-sectional areas. Microspheres A (**A-2**,**A-3**). Microspheres B (**B-2**,**B-3**). Microspheres C (**C-2**,**C-3**).

Using the methods established and optimized above, the microspheres produced by three manufacturers were analyzed. The pore structure of the microspheres’ surface and cross-sections was characterized by the SEM-DLIS technique, as shown in Figure 4. This approach enabled the quantitative analysis of pore number, pore area proportion, and pore size distribution (based on pore diameter) for each cross-section, as detailed in the methodology (Section 2.3.1, Section 2.3.2, Section 2.3.3 and Section 2.3.4) and summarized in Figure 5. To assess spatial heterogeneity, each cross-section was divided into three regions of equal area (Figure 6). The porosity, pore area proportion, and pore uniformity within each region was calculated, and the results are presented in Table 2.

The quantitative analysis corroborated the morphological observations described in Section 2.2, consistent with the SEM images. Microspheres A exhibited a non-porous surface. Analysis of the cross-sections (Figure 4A-II,A-III; Figure 5a-2,a-3) revealed a similar pore distribution pattern between large and small particles. The pore density (pores/mm^2^) was highest in the 0.0–0.3 μm and 0.3–0.6 μm size ranges. In terms of the total pore area, however, the 0.3–0.6 μm and 0.6–0.9 μm ranges were dominant, each contributing approximately 40% of the total porosity. As summarized in Table 2, the cross-sectional porosity was higher in the small particles (19.79%) than in the large particles (11.79%), although both exhibited comparable uniformity.

The pore structure of Microspheres B, including pore density, size distribution, and area proportion, differed markedly between its surface and the cross-sections of large and small particles (Figure 4B-I–B-III; Figure 5b-1–b-3). Surface porosity was significantly lower than internal porosity. Cross-sectional analysis revealed that large particles contained a mixture of large and small pores, with nearly equal area contributions from each. In contrast, small particles were dominated by a high density of sub-0.3 μm pores (5.74 million counts/mm^2^), a value substantially greater than that in large particles (0.25 million counts/mm^2^). As quantified in Table 2, the overall porosity of large particles (13.20%) was significantly lower than that of small particles (25.96%).

Microspheres C exhibited a surface characterized by numerous large pores (Figure 4C-I; Figure 5c-1). The internal pore distribution in its large particles (Figure 4C-II; Figure 5c-2) was similar to that of Microspheres A, being predominantly composed of small pores. In contrast, the small particles of Microspheres C showed a high proportion of large pores by area (Figure 4C-III; Figure 5c-3). Despite this marked difference in internal pore architecture, the overall porosity of both the large and small particles was comparable, at approximately 12–13% (Table 2).

### 2.4. Drug Distribution Study

LCRS enables the characterization of API distribution within microspheres through non-destructive, in situ, 3D imaging. A primary requirement for this technique is that the laser must penetrate the sample. For most non-transparent materials, laser penetration is limited; the penetration depth is inversely proportional to the material’s absorption coefficient (*k*) and directly proportional to the laser wavelength (*λ*, penetration depth = *λ*/4*πk*). It should be noted that even if the sample is non-absorptive, sample uniformity is also crucial for LCRS, as inhomogeneity may cause elastic scattering which can interfere with laser penetration and signal collection. In addition, any interfaces (such as air/microsphere and microsphere/API interfaces) can also cause laser reflection. When the laser beam passes through such media in a specific direction and reaches a certain depth, these reflections will reduce the laser power density, further affecting the effectiveness of laser penetration and the accuracy of subsequent detection. PLGA, as a drug carrier in microspheres, exhibits physical properties that satisfy this critical requirement. PLGA-based microspheres are optically transparent, analogous to glass beads, allowing the laser to pass through and be focused on any internal point for analysis. Once this prerequisite is met, optimizing the sensitivity and spatial resolution of the imaging process becomes essential for obtaining accurate analytical results. Therefore, meticulous optimization of all key instrumental parameters involved in the measurement is crucial.

#### 2.4.1. Wavelength Selection

**Figure 7:**
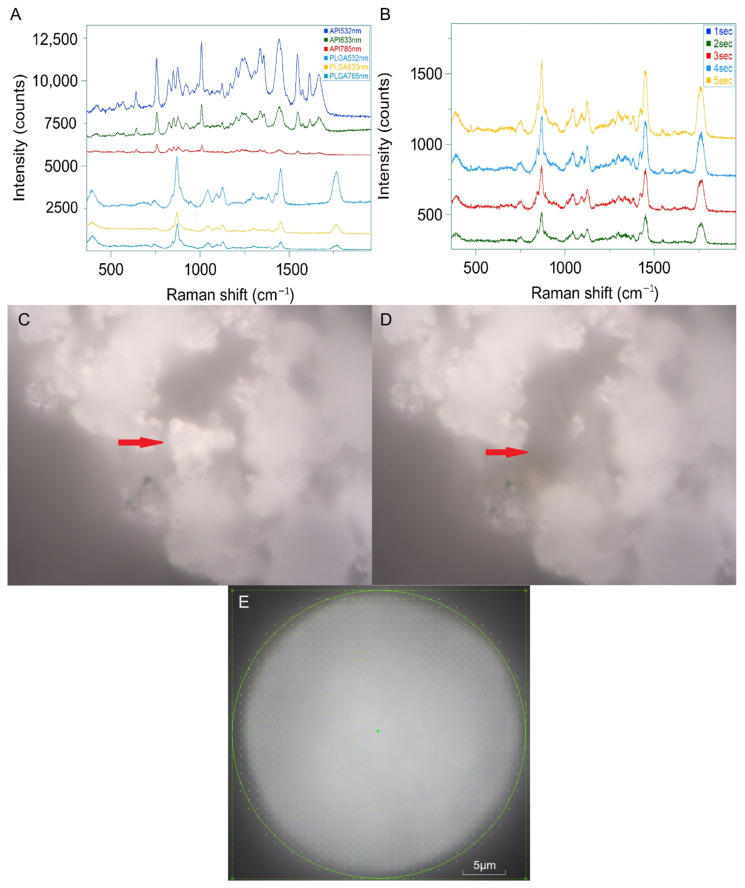
Spectra of API and PLGA at different wavelengths (**A**). Spectra were collected from 1 to 5 s (**B**). API burned before (**C**). API burned after 12-s spectral collection (**D**). Collection area setting (**E**). The position indicated by the arrow is the location where sample burning occurred before and after sampling in the same field of view.

The selection of an appropriate laser wavelength is a critical initial step. Commonly available laser sources for Raman spectroscopy include wavelengths of 532 nm, 633 nm, and 785 nm. Detector sensitivity and the reflectivity (diffraction efficiency) of diffraction gratings are both wavelength-dependent. Raman signal intensity is closely associated with the excitation wavelength, and is inversely proportional to the fourth power of the laser wavelength. For this reason, the Raman signal intensity acquired at 532 nm is generally higher than that at 785 nm. A longer wavelength provides greater laser penetration depth but yields lower signal intensity (due to reduced photon energy). Conversely, a shorter wavelength offers higher sensitivity but with a trade-off of reduced penetration and a higher risk of fluorescence interference. Fluorescence interference is a significant concern; when the wavelength of the scattered light is close to the sample’s native fluorescence emission, the resulting background can overwhelm the weaker Raman signal, severely compromising data quality. To identify the optimal wavelength, Raman spectra of the pure API and PLGA were collected at 532 nm, 633 nm, and 785 nm (Figure 7A). The 532 nm laser was selected for subsequent measurement as it provided the best signal-to-noise ratio by minimizing fluorescence interference while maintaining sufficient penetration for analysis.

#### 2.4.2. Diffraction Grating Selection

The selection of the diffraction grating is vital, as the number of grooves per millimeter (gr/mm) directly determines the spectral resolution; a higher groove density provides greater resolution. However, higher spectral resolution reduces the number of photons incident on each detector pixel. A reduced photon count at the detector further decreases the effective signal intensity from the sample. When the detector’s dark noise, other background noise, and artifact levels remain constant, this results in an elevated limit of detection compared with lower-resolution configurations. The Raman spectrometer with diffraction grating is typically equipped with gratings of 600, 1200, and 1800 gr/mm. A grating with a high groove density (e.g., 1800 gr/mm) is essential for distinguishing Raman peaks in compounds with close frequencies. Even for substances with highly similar molecular structures, their vibrational spectra can still exhibit obvious differences. Molecular crystals composed of identical molecular units often show remarkable discrepancies in the low-frequency external vibration region. Isotopically substituted and unsubstituted compounds share exactly the same crystal structure, yet their vibrational mode frequencies differ substantially. Additionally, distinct compounds may display spectral peak overlap within certain characteristic frequency ranges. If such overlap occurs for modes with high Raman activity, it will further complicate the qualitative identification of substances. However, since PLGA and the API have distinctly different chemical structures, a 600 gr/mm grating was selected. This choice prioritizes high signal intensity to ensure robust detection of both components without sacrificing the resolution necessary to clearly resolve their characteristic peaks.

#### 2.4.3. Objective Lens Magnification Selection

The selection of the objective lens magnification is important for determining spatial resolution. When the laser is focused through the objective, it forms a diffraction-limited spot at the measurement point. The size of this spot is inversely proportional to the spatial resolution; a smaller spot yields higher spatial resolution. The instrument is typically equipped with 20×, 50×, and 100× objectives. Under 532 nm laser excitation, the 50× (NA = 0.5) and 100× (NA = 0.9) objectives, which produce spot sizes of approximately 1 μm and 0.7 μm, respectively, are suitable for micro-scale analysis. To maximize the spatial resolution for mapping the internal distribution of the API within the microspheres, a 100× objective lens was selected for all measurements in this study.

#### 2.4.4. Confocal Pinhole Selection and Signal Separability at Specific Depths

The selection of confocal pinhole diameter is critical for balancing axial resolution and signal intensity. To optimize this parameter, calibration experiments were performed using a standard silicon wafer. Measurements were conducted using pinhole diameters of 50 μm and 500 μm, respectively. The corresponding confocal ratio, defined as the ratio of the signal intensity at the focal plane to that under non-confocal conditions, reached 83.3%. This result quantitatively demonstrates that the pinhole diameter directly governs the number of collected photons. A smaller pinhole effectively blocks out-of-focus light, allowing the system to achieve improved spatial resolution while preserving the majority of signal intensity (>80%).

In addition, the feasibility of layer-resolved signal separation along the *z*-axis was validated through three-dimensional imaging of microspheres. The cross-sectional diameter of the microsphere changes regularly with depth (*z*-axis), which is consistent with the actual spherical geometry of the particle. Signal intensity maps acquired at different depths accurately reflect these morphological variations, confirming that signals originating from distinct focal planes can be effectively isolated. Insufficient axial resolution would result in a three-dimensional reconstruction unable to reproduce the spherical structure of the microsphere. These results verify that the selected pinhole configuration enables precise depth-resolved analysis, which is essential for reliable three-dimensional chemical mapping.

#### 2.4.5. Measurement Protocol Optimization

The final step in optimizing the measurement protocol involves determining the signal collection time. Laser energy is highly concentrated, and prolonged spectral acquisition may cause sample degradation. Increasing the collection time (*t*) raises the total energy dose, which can elevate the local temperature and potentially cause thermal damage to the sample, as illustrated in Figure 7C,D. The spot area influences the laser power density and consequently regulates thermal effects. For 3D imaging, data must be acquired layer-by-layer at different depths (*z*-axis) according to a predefined step size. Sufficient acquisition time must be allocated for each point, especially at greater depths where signal intensity may be lower, because elastic scattering caused by sample inhomogeneity and reflections at interfaces such as air–microsphere and microsphere–API reduce laser intensity. Accordingly, the laser power density declines as the laser penetrates deeper into the medium along a given direction to ensure accurate signal detection. Collection efficiency is a major practical consideration. Using a microsphere with a diameter of approximately 30 μm as an example, the step sizes for the *x*, *y*, and *z*-axes were set to 1 μm to achieve a balance between spatial resolution and data volume for statistical significance. The collection area (Figure 7E) was defined to match the size of the microspheres, resulting in approximately 1000 collection points per layer. The total acquisition time for a single layer is therefore 1000 × *t*, and for the entire microsphere (e.g., 30 layers), it becomes 1000 × *t* × 30. Given the extended total acquisition time and the finite lifespan of the laser, whose intensity can decay over time, it is crucial to determine the minimum per-point collection time that still provides adequate signal strength. To establish this optimum, a test point on the deepest layer of a representative microsphere was selected, and spectra were acquired using different collection times (Figure 7B). The shortest time yielding a spectrum with an acceptable signal-to-noise ratio was selected for all subsequent measurements.

#### 2.4.6. Characteristic Spectral Peaks Selection

**Figure 8:**
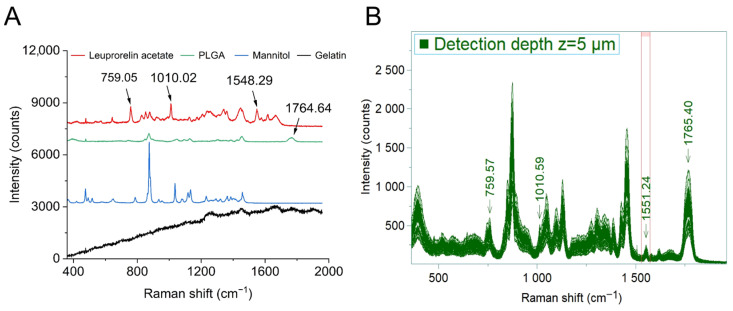
Raman spectra of different components (**A**). Mapping spectrogram (**B**).

Following the optimization of instrumental parameters, Raman spectra were collected for the pure components: the API, PLGA, and the excipients mannitol and gelatin. Characteristic peaks with strong intensity and minimal spectral overlap were identified for each component (Figure 8A). The selected target peaks for subsequent imaging were 1764.64 cm^−1^ for PLGA and 1548.29 cm^−1^, 1010.02 cm^−1^, and 759.05 cm^−1^ for the API. For the final imaging analysis, a subset of these peaks was chosen to ensure specificity. Due to potential interference from neighboring peaks near 1010.59 cm^−1^ and 759.57 cm^−1^, the API peak at 1551.24 cm^−1^ and the PLGA peak at 1765.40 cm^−1^ were selected as the most unambiguous markers. The intensities of these two peaks were used for the chemical mapping across different layers of the microsphere (Figure 8B). The vibrational assignments of all peaks are listed in Table A3 in Appendix A.

**Figure 9:**
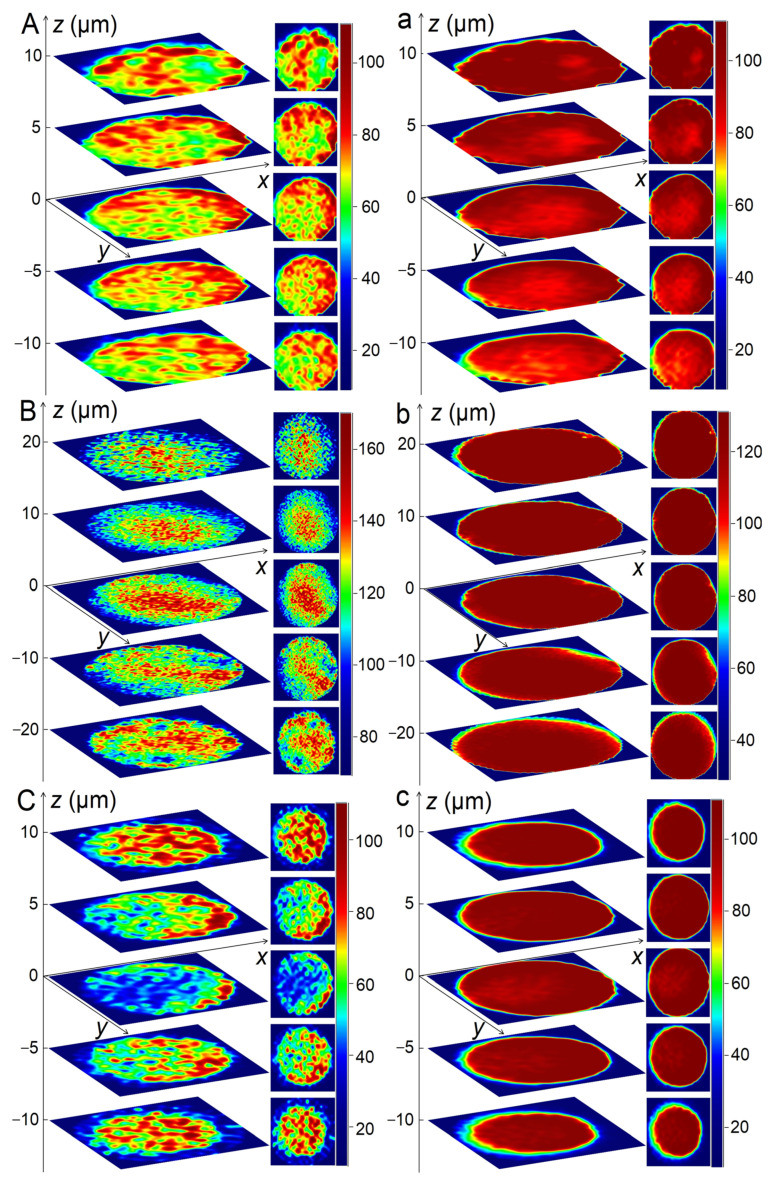
Heatmap of API and PLGA distribution, where red indicates high API concentration, blue indicates low concentration, and green represents intermediate levels. Microspheres A (**A**,**a**). Microspheres B (**B**,**b**). Microspheres C (**C**,**c**).

LCRS was employed to generate chemical distribution heatmaps for the API and PLGA within the patented and two modified leuprorelin acetate microspheres (Figure 9). This technique provides a visual representation of component distribution. As the matrix carrier, PLGA constituted the vast majority (>90%) of the microspheres’ mass and was uniformly distributed throughout all formulations (Figure 9a–c). In contrast, the API distribution varied significantly. In Microspheres A (Figure 9A), the API was uniformly dispersed from the core to the surface. Microspheres B (Figure 9B) exhibited a relatively even internal API distribution, with a tendency toward central accumulation and a moderate presence near the surface. Conversely, Microspheres C (Figure 9C) showed a pronounced concentration of API primarily within the outer layer. These distinct API distribution patterns were visually evident from the surface characteristics of the heatmaps: Microspheres A displayed a uniform green surface, while both B and C showed irregular blue areas on their surfaces, indicating less API at the periphery. The API distribution patterns revealed by LCRS correspond directly to the microstructural characteristics observed by SEM (Figure 3), providing a coherent explanation for the differing drug release profiles.

The LCRS imaging results revealed significant differences in the three microsphere formulations, which were reflected in their distinct internal API distributions and surface characteristics. Specifically, Microspheres B exhibited a broad particle size distribution (Figure 1, Table 1). Its internal structure, characterized by a dense core with minimal porosity and increasingly larger pores toward the surface, resulted in a relatively uniform, internally concentrated API distribution (Figure 9B). In contrast, Microspheres A underwent a unique heating process that reduced surface porosity. This was visualized as fewer blue areas (indicating low API concentration) on its surface in the LCRS image (Figure 9A). Combined with its narrow particle size distribution (Figure 1, Table 1), this led to a highly uniform API dispersion throughout the microspheres. Although Microspheres C shared a similar particle size distribution with A (Figure 1, Table 1), its unique manufacturing process created a highly porous structure from the core to the surface, with some particles embedding within these pores. The LCRS image for Microspheres C (Figure 9C) comprehensively captured this: the red color (high API concentration) was predominantly distributed in the outer layer, with distinct “dot-like” red areas at the edges. This pattern corresponded directly to the surface features observed in the SEM image (Figure 3C-1), confirming that the API was primarily located on the microspheres’ exterior.

**Figure 10:**
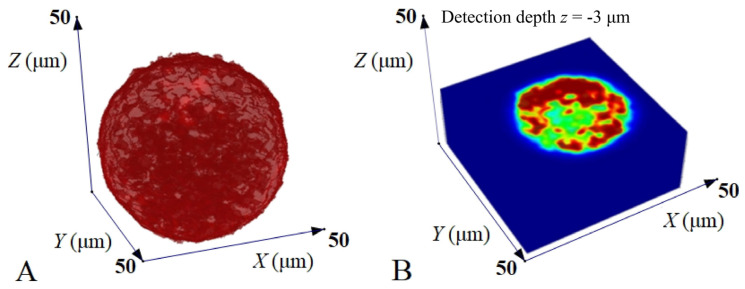
LCRS 3D animation of API distribution for Microspheres A (**A**). Display of API distribution layer by layer for Microspheres A (**B**).

Using LCRS imaging technology in conjunction with LabSpec 6 software, the acquired spectral data can be reconstructed into 3D visualizations. These models provided an intuitive representation of the internal distribution of the API and PLGA within the microspheres. The spatial concentration gradient of a component was represented by color intensity or depth, as shown in the 3D projection in Figure 10A. Furthermore, the distribution can be analyzed layer by layer, allowing for detailed examination of the API concentration at specific depths within the microsphere, as illustrated in the cross-sectional view in Figure 10B.

## 3. Discussion

This study successfully established two novel characterization methods, SEM-DLIS and LCRS, enabling precise quantification of the microphysical structure (surface and internal pore structure) of microspheres, as well as non-destructive visualization and in situ quantification of the spatial chemical distribution of internal drugs. Based on this, the “process–structure/distribution–performance” relationship of three leuprorelin acetate microsphere formulations were systematically investigated, providing definitive microscopic evidence for understanding the mechanisms underlying their release differences.

### 3.1. Microstructure as the Determinant of Differences in In Vitro Release Behavior

Our study indicates that the core factors determining release behavior extend beyond traditional macroscopic metrics and lie in deeper-level microstructural attributes. Specifically, the surface structure governs the initial burst release, while the internal structure and drug spatial distribution jointly regulate the sustained release kinetics.

While the current study establishes a strong empirical correlation between microstructural attributes (porosity, API localization) and release kinetics, we acknowledge that the development of a theoretical mathematical model is warranted for rigorous validation. Future work will focus on integrating these high-dimensional characterization datasets into mechanistic release models or AI-driven predictive algorithms to deconvolute the complex interplay between particle size, pore connectivity, and spatial drug distribution.

#### 3.1.1. Surface Porosity and API Surface Enrichment Synergistically Govern Initial Burst Release

The initial 1 h release rates of the three microspheres (A: 9% < B < C: 13%) closely followed the order of surface porosity quantified by SEM-DLIS (A: 0%, B: 10.97%, C: 8.31%). However, the release kinetics cannot be explained by porosity or API distribution in isolation; rather, it is the coupling of these two factors that dictates the behavior. While SEM-DLIS quantified a significant surface porosity of 10.97% in Microspheres B, LCRS imaging (Figure 9C) revealed that the API concentration at the surface of Microspheres C exceeded that at its core by greater than 3-fold (surface-to-core ratio > 3). This co-localization of the drug reservoir within the open porous network creates a synergistic “dual-acceleration” effect, maximizing the surface area for medium ingress and dissolution, thereby explaining why Microspheres C achieved the highest initial burst (13% at 1 h) despite its moderate particle size (Dv (50) = 17.1 μm). Conversely, Microspheres A (Figure 9A) exhibited a uniform API distribution (LCRS radial profile CV < 5%), but its surface porosity was negligible (0%, SEM-DLIS). This decoupling between drug presence and transport channels restricted its initial burst to only 9%, completely offsetting the theoretical advantage of its smaller particle size (Dv (50) = 15.3 μm).

#### 3.1.2. Internal Pore Structure, Particle Size, and Overall API Distribution Jointly Regulate Sustained Release Kinetics

During the sustained release phase, drugs must diffuse through a complex internal pore network. The interplay between pore connectivity (SEM-DLIS) and API spatial distribution (LCRS) overrides the theoretical effect of particle size differences.

Microspheres A (Dv (50) = 15.3 μm) had the smallest particle size and theoretically should release the fastest. However, its internal pores were predominantly sub-micron in size with relatively low porosity (11.79% for large particles, Table 2), and the API was uniformly distributed (Figure 9A). This dense internal structure and decoupled drug distribution resulted in the slowest sustained release rate.

Microspheres B (Dv (50) = 51.1 μm) had the largest particle size and theoretically should release the slowest. Yet, its higher internal porosity (13.20% for large, 25.96% for small particles, Table 2) and larger pore sizes (0.6–1.8 μm, Figure 5b-2,b-3) provided a more permeable network. The coupling of this porous connectivity with a slight central accumulation of API (LCRS) enabled its release rate to surpass that of Microspheres A. Its API distribution, slightly concentrated toward the center (Figure 9B), also indicates that the drug’s outward diffusion from the center is effectively supported by the pore structure.

Microspheres C had a particle size similar to A, but its API was highly enriched in the outer layer (Figure 9C). This “drug distribution advantage”, coupled with rapid medium ingress via surface macropores, collectively led to its fastest sustained release rate.

These observations underscore that the interaction between structural connectivity and chemical spatial distribution, rather than any single factor in isolation, is the fundamental determinant of transport phenomena.

Pore uniformity analysis (homogeneity index *Hs* > 88%, Table 2) indicated that the internal pores of the three microspheres were relatively uniformly distributed across the cross-sections. This provides important support for reasonably extrapolating the overall 3D structure of the microspheres from limited two-dimensional cross-sectional analysis, and also suggests that the process control over the macroscopic uniformity of internal pores is relatively stable.

### 3.2. Depth-Resolved API Distribution and Its Impact on Release Kinetics

Beyond the planar distribution, the vertical (*z*-axis) homogeneity of the API, as revealed by LCRS 3D imaging, provides critical insights into the diffusion pathways. However, the axial distribution alone cannot fully explain the release kinetics; its coupling with the internal pore network is equally decisive.

The depth-resolved profiles (Figure 9A–C) revealed distinct vertical stratification patterns among the formulations, which must be interpreted in conjunction with their microstructural porosity (Table 2).

Microspheres A exhibited a consistent API signal intensity across different z-layers, indicating a homogeneous core-to-surface distribution along the axial direction. This uniform axial distribution, coupled with its low internal porosity (11.79% for large particles) and zero surface porosity, minimized the initial concentration gradient but restricted the sustained diffusion rate, resulting in the slowest overall release.

Microspheres B showed a slight axial gradient, with the API signal tending to concentrate toward the core (lower z-values). This “inner-accumulated” vertical profile, coupled with its higher internal porosity (25.96% for small particles) and moderate pore sizes (0.6–1.8 μm), created a balanced diffusion scenario—the drug diffused steadily from the dense core through the permeable matrix, aligning with its intermediate sustained release rate.

Microspheres C displayed a pronounced axial heterogeneity, with the highest API concentrations localized in the upper layers (peripheral regions). This “outer-enriched” axial stratification, coupled with its surface macropores (8.31% surface porosity) and porous core-to-surface structure, drastically shortened the diffusion path length for the majority of the API, explaining its rapid release profile despite a particle size similar to Microspheres A.

These depth-resolved observations further confirm that the axial spatial coordinates of the API (combining radial and vertical distribution) must be evaluated alongside the pore connectivity metrics to fully elucidate the transport phenomena. The 3D spatial architecture—defined by the interplay of porosity, radial distribution, and axial stratification—dictates the effective surface area for dissolution and the diffusion distance, which are the primary physical determinants of the observed release kinetics.

### 3.3. The Role of Process Parameters in Shaping Microstructural Diversity

All observed differences in morphology, pore structure, and drug distribution were strongly correlated with inherent differences in the CPPs during preparation. This provides evidence in favor of the core hypothesis that “process determines structure”.

The non-porous surface of Microspheres A is likely attributed to a heating step in its production process that likely exceeded the glass transition temperature (Tg) of PLGA, causing surface polymer melting and pore sealing.

The unique surface macropores, embedded API crystals in Microspheres C (Figure 3C-1), and the API outer-layer enrichment confirmed by LCRS (Figure 9C) can be explained by its specific emulsification, curing, and lyophilization processes. This process promotes the migration of API molecules from the inner aqueous phase toward the oil/water interface, where they become entrapped within the polymer surface layer and pores during curing.

CPPs such as oil phase viscosity, stirring rate, curing temperature, and duration precisely “design” the final product’s pore size, distribution, and API migration path and final location by controlling droplet formation, solvent evaporation, and phase separation kinetics. Differences in pore structure between particles of different sizes (e.g., the disparity in porosity between large and small particles of Microspheres B) also stem from the inherent influence of droplet size on interfacial tension and mass transfer kinetics during curing [29].

### 3.4. Methodological Innovations and Implications

#### 3.4.1. Methodological Innovations: Complementarity, Non-Destructiveness, and In Situ Analysis

The two characterization methods, SEM-DLIS and LCRS, established in this study offer significant methodological advantages.

Firstly, as a complementary technology, SEM-DLIS provides high-throughput, precise quantification of physical structure, while LCRS provides spatial distribution and 3D visualization of chemical composition. Their combination enables a comprehensive analysis of the “physical structure–chemical distribution” of microspheres, with synergistic effects far exceeding those of any single technique. For instance, while SEM infers pores as release channels, LCRS directly confirms the enrichment of drugs near these pores, thereby fully validating the release mechanism.

Secondly, non-destructive and in situ analysis can be conducted. Utilizing the laser transparency of the PLGA matrix, LCRS technology achieves in situ, 3D scanning of intact microspheres internally, without any physical sectioning or chemical treatment that might alter the sample’s microstructure, ensuring the authenticity of the analysis.

#### 3.4.2. Methodological Advantages, Applicability, and Limitations

While the SEM-DLIS method offers high-throughput quantification, its accuracy relies on the quality of SEM image segmentation; artifacts from sample preparation (e.g., ion beam polishing) could potentially affect pore boundary recognition. Regarding LCRS, although the 532 nm laser provides high resolution, it may induce thermal degradation in light-sensitive APIs; thus, careful power calibration is required. Furthermore, the current LCRS 3D reconstruction is based on discrete *z*-axis layers; future integration with continuous depth profiling could enhance spatial resolution. Nevertheless, these methods are highly applicable to optically transparent polymeric microcarriers but may require adaptation (e.g., longer wavelengths) for highly opaque or carbon-based drug delivery systems.

#### 3.4.3. Profound Implications for Generic Drug Development and Quality Evaluation

The findings of our study provide clear guidance for the development of generic drugs for complex injectables like PLGA microspheres.

Firstly, establishing the centrality of microstructural and physicochemical equivalence. This strongly demonstrates that achieving consistency with the reference listed drug (RLD) only in composition and macroscopic indicators is insufficient to ensure bioequivalence (BE) [6,30]. The pursuit of equivalence in microstructure (porosity, pore size, distribution) and drug spatial distribution is essential. The characterization methods developed here provide a direct and reliable tool for assessing microstructural and physicochemical consistency.

Secondly, providing a precise benchmark for reverse engineering and process control. Generic drug developers can use this system to reverse analyze the microstructural and physicochemical attributes of the RLD and then forward-design the process, precisely identifying and controlling the CPPs that influence these attributes. This facilitates the transition from “quality by testing” to “quality by design (QbD)” [31].

Finally, addressing gaps in existing quality standards. Current quality standards generally lack control items for microstructure. The characterization methods and indicators established in this study can provide scientific basis and technical support for improving the quality standards of complex injectables and enhancing product quality controllability.

#### 3.4.4. Implications for Innovative Drug Research and Development

This study also holds significant value for innovative drug development. By characterizing the pore structure and drug distribution of microspheres under different formulation processes, a clear link between “formulation/process–microstructure-release behavior” can be established. This provides explicit microscopic guidance and a decision-making basis for the rational optimization of formulations and the precise modulation of release rates (e.g., controlling burst release, extending efficacy), thereby accelerating the product development process.

## 4. Materials and Methods

### 4.1. Materials

Leuprorelin acetate microspheres for injection were sourced respectively from Takeda Pharmaceutical Company Limited (Osaka, Japan), Beijing Boente Pharmaceutical Company Limited (Beijing, China), and Shanghai Livzon Pharmaceutical Company Limited (Shanghai, China). PLGA 752H (Mw = 14 kDa, L/G = 75/25, free-acid terminated) was obtained from Evonik (Birmingham, AL, USA). Mannitol and gelatin were purchased from U. S. PHARMACOPEIA (North Bethesda, MD, USA). Leuprorelin acetate was supplied by Shanghai Lizhu Pharmaceuticals (Shanghai, China). Acetonitrile and acetic acid (HPLC grade) were acquired from Merck KGaA (Darmstadt, Germany). All other chemicals were of analytical grade and obtained from commercial suppliers.

### 4.2. Determination of Drug Loading

The drug loading was quantified using HPLC. Briefly, 20 mg of microspheres were accurately weighed and transferred to a 10 mL volumetric flask. Then, 2 mL of acetonitrile was added, and the mixture was sonicated until the microspheres were completely dissolved. Subsequently, approximately 0.4 mL of a 0.1% (*v*/*v*) acetic acid solution was added slowly with shaking until the solution clarified. The solution was then diluted to volume with 0.1% (*v*/*v*) acetic acid and mixed thoroughly. An aliquot of the solution was centrifuged at 13,000 rpm for 10 min. The supernatant was analyzed using an Waters 2695 photodiode array detector (Waters, Milford, MA, USA). Detection was performed at a wavelength of 220 nm. API was separated on an XBridge Peptide BEH C18 column (250 × 4.6 mm, 5 μm; Waters, Milford, MA, USA). A isocratic elution program was applied with acetonitrile and 0.5% (*v*/*v*) phosphoric acid (15:85, *v*/*v*). Flow rate was 1.5 mL/min, and the injection volume was 10 μL. The drug loading was calculated using the following equation:

Drug loading = (mass of drug entrapped/mass of microspheres) × 100% (7)

### 4.3. Determination of Particle Size Distribution

The particle size distribution of the leuprorelin acetate microspheres was determined using a laser diffraction particle size analyzer (Mastersizer 3000, Malvern Instruments Ltd., Malvern, UK). Approximately 100 mg of microspheres was dispersed in 100 mL of distilled water containing 0.1% (*w*/*v*) polysorbate 20. The analysis was performed with the following optical parameters: a particle refractive index of 1.49 (determined using an Abbe refractometer) and a dispersant (water) refractive index of 1.33 [32].

### 4.4. In Vitro Release

Based on literature reports and experimental optimization, a method for determining the in vitro release kinetics of leuprorelin acetate microspheres was established [33]. Briefly, approximately 10 mg of microspheres was suspended in 100 mL of release medium, which contained a specified concentration of surfactant (e.g., polysorbate 80), and incubated at 37 °C under constant agitation. At predetermined time intervals (1, 5, 9, 22, 24, and 48 h), 1 mL aliquots of the release medium were withdrawn. The drug concentration in these samples was quantified using the same HPLC method described in the drug loading analysis section.

### 4.5. Characterization of Morphology

Microsphere samples were immobilized in ultraviolet-cured resin. The embedded samples were then sectioned using a soft ion beam cryo-polishing instrument (SCP-100, JIT Instruments Co., Ltd., Yueqing, China) with a 3 kV argon ion beam at −30 °C. Prior to imaging, the sections were sputter-coated with a conductive metal layer to enhance surface conductivity and improve image quality. Finally, the cross-sections were imaged using a high-resolution field-emission scanning electron microscope (FE-SEM; Hitachi SU5000, Hitachi High-Tech Corporation, Tokyo, Japan).

### 4.6. Determination of Porosity, Proportion of Pore Area and Pore Size Distribution

Unlike traditional threshold-based methods, our approach utilized a CNN with an improved U-Net architecture for semantic segmentation. This model was trained on a pre-labeled dataset using supervised learning, optimized with a cross-entropy loss function, and enhanced with data augmentation strategies (e.g., rotation, scaling, contrast perturbation) to improve its generalization capability. This enabled the automatic and precise pixel-wise classification of SEM images into “pore” and “matrix” regions [34,35]. A key limitation of conventional porosity analysis is its reliance on 2D image statistics, which only provide projections and fail to accurately represent the true 3D pore distribution on the curved surface of a microsphere. To overcome this, we introduced a pixel-level pore projection correction algorithm based on a spherical geometric model. This algorithm, applied after the DLIS, allows for a more accurate estimation of true surface porosity [36]. All image analysis was conducted in a Python environment. Model training and inference were performed using the PyTorch (v2.9.1, Meta Platforms, Inc., Menlo Park, CA, USA) framework, supplemented by the OpenCV and scikit-image libraries for image processing [37,38].

In this study, the connected component labeling algorithm was employed to convert the binary pore mask obtained from semantic segmentation into instance segmentation results. The detailed implementation is as follows:

(1) Connectivity definition: 8-connectivity was adopted in 2D cross-sectional images (i.e., each pixel is evaluated for connectivity with its adjacent pixels in the up, down, left, right, and diagonal directions) to ensure proper connection and separation of adjacent pore regions.

(2) Implementation tool: The automatic labeling of connected components was performed using the scipy.ndimage.label function in Python (v3.13.2, Python Software Foundation, Beaverton, OR, USA).

(3) Noise filtering: To eliminate potential noise or isolated artifacts from image segmentation, a minimum pore volume threshold of 5 voxels (corresponding to a physical area of approximately 5 × (0.2 μm)^2^ = 0.2 μm^2^) was set. Regions below this threshold were excluded from the subsequent size distribution statistics.

(4) Processing of adhered pores: No obvious adhesion between pore phases was observed in any of the images used in this study. Therefore, over-segmentation algorithms such as the watershed algorithm were not applied, and direct connected component labeling was sufficient to meet the separation requirements.

Furthermore, to ensure the consistency and comparability of pore extraction results across different images, a grayscale standardization procedure was implemented during the image acquisition stage: prior to each SEM imaging session, grayscale calibration was performed using standard samples of copper (grayscale reference value: 180) and aluminum (grayscale reference value: 60), guaranteeing that the grayscale levels of all images possessed consistent physical meanings. This procedure effectively improved the repeatability of the deep learning model segmentation results and provided a guarantee for the accurate identification of subsequent connected components.

The pre-annotated dataset used in this study consists of SEM images of microspheres with particle sizes ranging from 0 to 500 μm, sourced from multiple manufacturers and formulated with various materials including PLGA, PCL, etc. The total dataset size was approximately 200 TB with a large volume of image data. All images were processed following the aforementioned grayscale standardization procedure during acquisition to guarantee data quality. Each image was manually annotated for pore contours by multiple materials scientists using the open-source LabelMe tool (MIT CSAIL, Cambridge, MA, USA), and all annotations were cross-validated for quality control.

### 4.7. Determination of Drug Distribution by LCRS

All measurements were performed using a laser confocal Raman spectrometer (LabRAM HR Evolution, HORIBA Scientific, Kyoto, Japan) equipped with a 532 nm Nd:YAG (neodymium-doped yttrium aluminum garnet) laser. The laser power at the sample was attenuated to approximately 50 mW using a neutral density filter. A 100× (NA = 0.9) objective lens with a confocal pinhole aperture set to 50 μm was used to focus the laser onto a single microsphere, yielding a laser spot radius of approximately 0.7 μm. Raman scattering signals were detected by a charge-coupled device (CCD) cooled to −70 °C. Spectra were acquired over a wave number range of 400 to 2000 cm^−1^ using a diffraction grating with 600 gr/mm, with an acquisition time of 3 s and 1 accumulation. Raman spectra of the pure components, including leuprorelin acetate, PLGA, mannitol, and gelatin, were first collected under the same instrumental parameters. Characteristic peaks for each compound were identified, ensuring that the selected peaks exhibited no spectral overlap, and their precise peak positions were determined.

A 3D mapping procedure was employed to characterize the internal structure of individual microspheres. The equatorial plane (the cross-section with the maximum radius) was designated as the *z*-axis origin (*z* = 0 μm). From this plane, two additional measurement planes were selected at equidistant intervals above and below, resulting in a total of five parallel planes for analysis. For example, for a microsphere with a radius of 15 μm, measurements were taken at *z*-heights of +10 μm, +5 μm, 0 μm, −5 μm, and −10 μm. For each measurement layer, the scanned area was set to match the area of the maximum radius cross-section. The step size for both the *x* and *y* axes was set to 1 μm. All acquired Raman spectra were preprocessed using LabSpec 6 software (v6.7.1.10, Horiba Scientific, Kyoto, Japan), which included despiking (for cosmic ray removal) and baseline correction using a polyline fitting algorithm.

To ensure the representativeness of the Raman mapping results, microspheres with diameters close to the volume median diameter (Dv50) were selected as typical samples according to particle size distribution analysis. Microspheres with intact spherical morphology, no obvious breakage or adhesion were chosen under microscopic observation. For each formulation, 3–5 representative microspheres were analyzed to verify the repeatability of the API distribution patterns.

## 5. Conclusions

This study successfully established two complementary characterization methods: SEM-DLIS and LCRS. SEM-DLIS enabled the precise quantification of the microsphere microstructure, including surface and internal pore characteristics (such as porosity and pore size distribution), while LCRS achieved the non-destructive, in situ, 3D visualization and quantitative analysis of the spatial drug distribution within intact microspheres.

By applying these methods, the study revealed the following. (1) Significant differences exist in the microstructure of microspheres. Specifically, surface porosity ranged from 0% (Microspheres A) to 10.97% (Microspheres B), while internal porosity varied between ~12% and ~26%. Pore size distribution was predominantly in the 0.0–0.9 μm range, contributing over 80% of the total porosity. (2) The internal API distribution patterns varied markedly; Microspheres A showed uniform dispersion (green heatmap), B showed central accumulation, and C exhibited outer-layer enrichment (red heatmap).

The findings further elucidate the intrinsic determinants of the in vitro release behavior. The initial burst release (ranging from 9% to 13%) was governed by the synergistic effect of surface porosity and drug surface enrichment, whereas the sustained release kinetics were jointly regulated by the internal pore structure, particle size, and the overall spatial distribution of the drug. All observed microstructural differences may be attributed to the critical role of CPPs.

## Figures and Tables

**Table 1:** Drug loading and particle size distribution of the microspheres.

Name	Drug Loading (%)	Dv (10) (μm)	Dv (50) (μm)	Dv (90) (μm)	Dv (4,3) (μm)
Microspheres A	7.9	5.2	15.3	37.0	18.3
Microspheres B	8.2	14.7	51.1	97.5	54.8
Microspheres C	8.2	5.5	17.1	31.3	18.3

**Table 2:** Porosity, proportion of pore area, and uniformity in each region.

Microspheres	Figure	Porosity (%)	Region	Proportion of Pore Area (%)	Uniformity (%)
A	A-I	0	− ^1^		
	A-2	11.79	a1	10.28	89.56
			a2	12.66	
			a3	13.16	
	A-3	19.79	a1	18.68	92.75
			a2	20.46	
			a3	22.32	
B	B-I	10.97		−	
	B-2	13.20	a1	10.33	88.18
			a2	12.76	
			a3	13.80	
	B-3	25.96	a1	27.98	99.00
			a2	28.08	
			a3	28.62	
C	C-I	8.31		−	
	C-2	13.66	a1	17.69	90.67
			a2	17.47	
			a3	14.32	
	C-3	12.86	a1	11.46	81.62
			a2	17.87	
			a3	17.00	

^1^ No data.

## Data Availability

The original contributions presented in this study are included in the article. Further inquiries can be directed to the corresponding author.

## Abbreviations

The following abbreviations are used in this manuscript:

SEM	Scanning electron microscopy
DLIS	Deep learning-based image segmentation
LCRS	Laser confocal Raman spectroscopy
3D	Three-dimensional
API	Active pharmaceutical ingredient
PLA	Poly (lactic acid)
PLGA	Poly (lactic-co-glycolic acid)
FDA	Food and Drug Administration
EMA	European Medicines Agency
CPPs	Critical process parameters
CQAs	Critical quality attributes
CT	Micro-computed tomography
TTM	Transition temperature microscopy
EDS	Energy-dispersive X-ray spectroscopy
HPLC	High performance liquid chromatography
Dv	Volume-based particle size distribution
CNN	Convolutional neural network
*CV*	Coefficient of variation
*Hs*	Coefficient of variation
Tg	Glass transition temperature
RLD	Reference listed drug
BE	Bioequivalence
QbD	Quality by design
CCD	Charge-coupled device
CLS	Classical least squares
AVE	Average

## Appendix A

**Table A1:** Original data of particle size distribution.

**Microspheres A**					
− ^1^	Batch NO.			−	−
Dv/μm	522396	546605	547309	AVE/μm	SD/%
10	5.1	5.2	5.4	5.2	0.16
50	15.4	15.2	15.4	15.3	0.12
90	32.3	37.6	41.0	37.0	4.38
4.3	17.2	18.6	19.1	18.3	0.98
**Microspheres B**					
−	Batch NO.			−	−
Dv/μm	200104	221103	220408	AVE/μm	SD/%
10	15.0	14.2	14.8	14.7	0.42
50	47.6	54.6	51.2	51.1	3.50
90	88.9	110.0	93.7	97.5	11.06
4.3	50.4	61.5	52.4	54.8	5.92
**Microspheres C**					
−	Batch NO.			−	−
Dv/μm	210419	A240801	A240802	AVE/μm	SD/%
10	6.8	5.5	4.3	5.5	1.27
50	17.1	17.0	17.3	17.1	0.15
90	29.3	31.1	33.5	31.3	2.11
4.3	18.0	18.5	18.3	18.3	0.42

^1^ No data.

**Table A2:** Original data of in vitro release.

**Microspheres A**				
− ^1^	Batch NO.			Degree of release
Time/h	522396	546605	547309	AVE/%
1	9	8	9	8.7
5	34	29	30	31.0
9	45	40	46	43.7
22	73	76	79	76.0
24	76	79	82	79.0
48	93	95	97	95.0
**Microspheres B**				
−	Batch NO.			Degree of release
Time/h	200104	221103	220408	AVE/%
1	12	11	12	11.7
5	38	41	40	39.7
9	56	60	58	58.0
22	87	91	89	89.0
24	91	96	93	93.3
48	95	98	97	96.7
**Microspheres C**				
−	Batch NO.			Degree of release
Time/h	210419	A240801	A240802	AVE/%
1	13	14	13	13.3
5	43	47	46	45.3
9	77	81	80	79.3
22	85	89	88	87.3
24	88	91	91	90.0
48	92	94	96	94.0

^1^ No data.

**Table A3:** Wavenumbers and vibrational assignments of PLGA [39,40] and leuprorelin acetate [41,42,43].

Wavenumber, ν/cm^−1^	Assignments
PLGA	
1764.64, 1765.40	C=O stretching vibration
Leuprorelin acetate	
1548.29, 1551.24	Bending vibration of CH_3_, CH_2_ and CH
1010.02, 1010.59	C-N stretching vibration
759.05, 759.57	C=O in-plane scissoring vibration
